# Can routine health facility data be used to monitor subnational coverage of maternal, newborn and child health services in Uganda?

**DOI:** 10.1186/s12913-021-06554-6

**Published:** 2021-09-13

**Authors:** Geraldine Agiraembabazi, Jimmy Ogwal, Christine Tashobya, Rornald Muhumuza Kananura, Ties Boerma, Peter Waiswa

**Affiliations:** 1 Department of health policy planning and Management, Makerere University School of Public Health, Mulago New-Complex, Kampala, Uganda; 2 Ministry of Health, Kampala, Uganda; 3 Makerere University Centre of Excellence for Maternal, Newborn and Child Health, Mulago New-Complex, Kampala, Uganda; 4 Department of International Development, London School of Economics and Political Science, London, UK; 5 Institute for Global Public Health, University of Manitoba, Winnipeg, Canada; 6 Global Health Department of Public Health Sciences, Karolinska Institutet, Stockholm, Sweden

**Keywords:** Health facility data, District health information system, Data quality, Maternal health, Child health, Uganda

## Abstract

**Background:**

Routine health facility data are a critical source of local monitoring of progress and performance at the subnational level. Uganda has been using district health statistics from facility data for many years. We aimed to systematically assess data quality and examine different methods to obtain plausible subnational estimates of coverage for maternal, newborn and child health interventions.

**Methods:**

Annual data from the Uganda routine health facility information system 2015–2019 for all 135 districts were used, as well as national surveys for external comparison and the identification of near-universal coverage interventions. The quality of reported data on antenatal and delivery care and child immunization was assessed through completeness of facility reporting, presence of extreme outliers and internal data consistencies. Adjustments were made when necessary. The denominators for the coverage indicators were derived from population projections and health facility data on near-universal coverage interventions. The coverage results with different denominators were compared with the results from household surveys.

**Results:**

Uganda’s completeness of reporting by facilities was near 100% and extreme outliers were rare. Inconsistencies in reported events, measured by annual fluctuations and between intervention consistency, were common and more among the 135 districts than the 15 subregions. The reported numbers of vaccinations were improbably high compared to the projected population of births or first antenatal visits – and especially so in 2015–2016. There were also inconsistencies between the population projections and the expected target population based on reported numbers of antenatal visits or immunizations. An alternative approach with denominators derived from facility data gave results that were more plausible and more consistent with survey results than based on population projections, although inconsistent results remained for substantive number of subregions and districts.

**Conclusion:**

Our systematic assessment of the quality of routine reports of key events and denominators shows that computation of district health statistics is possible with transparent adjustments and methods, providing a general idea of levels and trends for most districts and subregions, but that improvements in data quality are essential to obtain more accurate monitoring.

**Supplementary Information:**

The online version contains supplementary material available at 10.1186/s12913-021-06554-6.

## Background

Household surveys are the most common and trusted source for monitoring service coverage in the population, but the frequency and sample size of surveys limits the ability to assess annual progress at national and subnational levels. Annual estimates of coverage are required to monitor national and local plans and programs for regular reviews which are used to adjust program priorities at national and subnational levels [1, 2].

Routine health facility data are potentially a useful source of more frequent estimates of service coverage at the local level, as has been shown in several countries [3–6]. Such facility-data derived estimates of coverage may focus on indicators of maternal, newborn and child health (MNCH), child immunization [7], malaria [8] and other infectious disease programs such as HIV and TB. In recent years, many countries have stepped up their investments in their routine health facility information systems, using a web-based data platform (District Health Information System, DHIS2) [9] leading to improvements in completeness of reporting and advances in data quality assessment [10, 11].

Data quality is often the greatest impediment to the use of routine health facility data for coverage estimates. The numerator of the coverage statistic may be affected by poorly designed or shortages of data collection instruments, recording errors, incomplete and inaccurate reporting [11–14]. WHO has published a set of methods and indicators to assess the quality of reported data on services which is available as a module within DHIS2, and can be used to detect and investigate outliers and inconsistencies [10, 15].

Coverage estimates are further hampered by the lack of accurate data for denominators or target populations such as births, especially for subnational units. Census population projections are the most common source of such denominators. If the most recent census was conducted several years ago, the likelihood that population projections are well-off the actual target populations increases. Geospatial analyses or alternative methods to improve target population estimates have been proposed [5, 16].

Uganda has a strong tradition of monitoring district performance to inform annual reviews. In 2003, the ministry of health introduced a system of annual district league tables which is still in use to assess district progress and performance as of today. The system currently uses an index based on 14 indicators of coverage, quality, human resources and reporting process, with RMNCH accounting for the largest share (six indicators) [17]. The system has been extensively reviewed, showing both the potential and limitations of district assessments with routine data [18, 19]. In addition, many countries including Uganda are increasingly using district scorecards based on routine facility data.

This paper examines the ability to generate statistics for selected MNCH coverage indicators at the subnational level using routine health facility data and assesses the extent to which such statistics can be used to monitor local progress.

## Methods

The focus is on districts as the main unit of health planning and program implementation in Uganda. Uganda’s 135 districts are grouped into 15 subregions and six regions. These (sub)regional classifications, however, are used for surveys such as the Uganda Demographic and Health Survey (UDHS) [20] but are not an operational part of the health system.

The routine health facility data are collected as part of service provision at all health facilities. The system is paper-based in most health facilities. Data are entered into the computer as part of a DHIS2 web-based software system at the district level. Compilation, quality check and initial analysis of the health facility data from the districts, are done by the Ministry of Health at the national level.

The Ministry of Health uses the population projections from the Uganda Bureau of Statistics (UBOS) within DHIS2 to obtain denominators for coverage estimates. The last population census was conducted on 27 August 2014. UBOS provides official district population projections for single years 2015–2020 including total population and population by single years and sex for districts, including for the new districts formed since the census. We also used the United Nations Population Division projections of total population and crude birth rates to estimate live births [21].

We analyzed the DHIS2 data for 2015–2019 at the national, sub-regional and district levels. The subregions have the advantage that the facility data derived coverage results can be compared with the results from the national surveys. In comparisons of facility data derived coverage estimates with survey results, the districts were given the same survey results as the subregion.

The most recent nationally representative population-based surveys were the UDHS 2016 and the Uganda Malaria Indicator Survey (UMIS) 2018/19 [22] which were used for external comparison and to obtain crude birth rates. The survey samples allowed for estimation of coverage indicators for the 15 subregions. The first antenatal visit (ANC1), first pentavalent (penta1) and BCG vaccinations had near universal coverage according to the Uganda DHS 2016: 97% of pregnant women, 95 and 96% of children 12–23 months respectively (Table 1). ANC1 coverage was equally high in the 2018/19 UMIS (96%) and all three indicators were well over 90% in the Uganda DHS 2011, indicating that these services have been used by nearly all Ugandan pregnant women and infants for a long period. Coverage was high in all 15 subregions in the UDHS for the three interventions. Therefore, assuming no decline in coverage for the three interventions, we expect that the numbers of ANC1 visits, penta1 and BCG vaccinations would increase exclusively because of population growth.

**Table 1:** General characteristics, crude birth rate per 1000 population (CBR), coverage (%) of antenatal care first visit (ANC1), first pentavalent vaccination (penta1) and BCG vaccination, Uganda DHS (UDS) and Uganda Malaria Indicator Survey (UMIS), by subregion

Subregion	Population 2015	Population growth rate (%)	Number of districts	CBR (UDHS 2016)	CBR (MIS 2018/9)	ANC1 (UDHS 2016)	ANC1 (UMIS 2018/9)	Penta1 (UDHS 2016)	BCG (UDHS 2016)
Acholi	1,535,100	2.8	8	38.0	34.6	97.3	99.0	98.7	96.9
Ankole	2,946,700	2.1	12	34.5	27.1	96.9	94.9	96.7	85.9
Bugisu	1,803,800	3.1	9	35.8	33.4	97.1	100.0	97.9	96.6
Bukedi	1,928,200	3.0	7	39.8	35.5	96.8	88.5	95.6	94.4
Bunyoro	2,102,300	4.3	8	42.3	39.7	92.3	98.6	94.4	87.7
Busoga	3,663,700	2.7	11	39.8	33.5	97.8	97.7	93.1	90.1
Kampala	1,528,800	1.7	1	38.2	28.1	97.9	96.9	94.8	92.3
Karamoja	991,600	3.3	9	45.5	49.3	97.3	88.9	98.5	96.5
Kigezi	1,390,500	1.2	6	32.0	34.1	99.8	99.4	98.3	96.2
Lango	2,111,400	2.9	9	35.2	33.5	97.1	99.0	95.5	95.4
North Central	3,773,500	2.7	12	37.5	29.3	98.8	90.3	92.0	86.0
South Central	4,474,500	3.9	13	39.9	29.2	95.8	94.9	90.9	84.7
Teso	1,870,600	3.3	10	43.0	38.0	98.9	98.5	97.9	96.6
Tooro	2,644,000	3.3	9	40.4	34.2	98.0	93.6	93.7	92.7
West Nile	2,727,400	3.0	11	40.8	34.4	98.7	98.8	97.6	94.5
National	**35,492,100**	**3.0**	**135**	**38.7**	**32.7**	**97.3**	**95.5**	**94.9**	**96.3**

We assessed the quality of the reported health facility data and resulting coverage estimates by considering (1) completeness of facility reporting (2) presence of extreme outliers (3) consistency of reported events over time; (4) consistency between reported numbers for antenatal care and immunization (5) assessment of target population estimates and (6) external consistency with survey coverage estimates, in line with the WHO Data Quality toolkit and related applications [5, 10, 11].

First, completeness of reporting of RMNCH events, which is reported on a single form, was based on the proportion of expected reports from all listed public, private-not-for profit and private health facilities. The extreme outliers were defined as at least 3.5 standard deviations from the expected value of data element for a particular year based on the median of the five-year period and were corrected if errors (e.g. an additional digit increasing the number by a factor 10) appeared the likely cause.

Second, consistency over time for ANC1, penta1 and BCG was assessed by comparing the reported numbers trend with expected numbers based on a log transformed regression analysis (ln(y) = ax + b, where y is the reported number and x the year). In case of the near-universal coverage interventions, the increases are only driven by population growth. We expected the slope a of the regression to be about 3% (within the range 1–4.9%) and the year-to-year fluctuations, as measured by the standard errors of the regression line, to be small if data quality is good.

Third, we assessed the internal consistency between interventions for immunization and antenatal care. The reported number of third doses of pentavalent vaccination (penta3) must be lower than that for penta1. We used the subregional ratios of the penta1 to penta3 vaccination coverage rates from the Uganda DHS 2016 by subregion to obtain an expected value for each subregion and district. We arbitrarily assumed that ratios within a range of plus or minus 15% (relative to the expected ratio), and a value not less than 1, presented plausible reporting of penta1 and penta3 vaccinations. The internal consistency of the reported numbers was further assessed by comparing annual ANC1 and penta1 reported numbers. As a reference, we used an expected ANC1 to penta1 numbers ratio of 1.11, ranging from 1.07 to 1.18 by subregion. This ratio was derived from the ANC1 and penta1 coverage rates in the UDHS 2016, and the loss of pregnancies between first ANC visit and first immunization after the neonatal period using the following assumptions: pregnancy wastage estimated at 8%, a twinning rate of 2% [23] and early infant mortality of 3 per 100 live births (based on the results of the UDHS 2016 [20]). Pregnancy wastage includes abortion between the first ANC visit (median 4.7 months of pregnancy duration in the UDHS 2016, estimated at 6%) and stillbirths (from end of the 7th month, estimated at about 2% [24])). We marked district and subregional ratios within the range of plus or minus 15% of the expected ratio as plausible (corresponding with at least five standard deviations).

Fourth, survey data, population projections and health facility data-based methods (5) were used to assess different denominators, and obtain the best estimate of the target populations, for the calculation of population coverage of interventions. Population projections are generally the primary source of denominators such as total population, live births or children eligible for immunization. We assessed the consistency of the population projections for live births, based on the UBOS total population projection and crude birth rates obtained from surveys (Table 1), with the estimated number of live births obtained from the reported number of events for near-universal coverage interventions of ANC1, BCG and penta1 vaccination. The computations were done for national, subregional and district level.

For the national level, we used a crude birth rate of 38.7 per 1000 population, obtained from the UDHS 2016 (and from StatCompiler for the subregions [25]), which we considered a more complete estimate than the UMIS 2018/19, given its larger sample and greater focus on the birth history data collection. The UDHS 2016 subregional crude birth rates were used for the subregions and districts (Table 1). In addition to the UBOS projections, we applied the United Nations Population Division population projections which estimated a population size which was 8% greater in 2015 and 10% greater in 2019 than the UBOS projection. The extent to which the use of population projections led to coverage estimates close to the expected coverage value for near-universal interventions of ANC1, penta1 and BCG was evaluated for districts and subregions, considering the absolute difference from the expected coverage as an indication.

Finally, we computed coverage rates for five interventions (four or more ANC visits, IPT 2 for pregnant women, institutional delivery care, third pentavalent and measles vaccination) using two methods of estimating the denominators: the population projections, using the UNPD data, and a health facility data derived denominator. The latter was computed from ANC1 reported numbers for the ANC4, IPT2 and institutional delivery indicators, and from penta1 for penta3 and measles vaccination indicators. The denominators include a small adjustment for non-coverage, based on the results of the most recent survey (e.g. if coverage was 95% add 5% for non-coverage), and take into account pregnancy loss, multiple births and early infant mortality as described above.

We assessed the performance of the two methods by assessing the difference with the survey values (UDHS except UMIS 2018/19 also for IPT2) and the variation over time. This included a comparison of the results for 2016–17 with the UDHS 2016, assuming that smaller differences indicate better data quality, while recognizing that coverage may have changed in the year following the UDHS 2016 for these interventions and that most survey data are retrospective. Absolute fluctuations greater than 20% of the previous year were considered less plausible.

## Results

Table 2 summarizes the different methods and results on the different dimensions of data quality.

**Table 2:** Summary of data quality measures at national, subregional and district levels, Uganda, 2015–2019

Data quality measure	Selected definitions and thresholds	National	Subregions	Districts
Completeness of facility reporting	Monthly facility MCH reports received out of total expected MCH reports	98%	> 95% in all years 2015–2019, with 4 exceptions	8/135 districts below 90% in 2018 and 2019
Extreme outliers	More than 3.5 SD from expected value	None	None	2 district values adjusted for a single year
Consistency over time	ANC1, expected 3% annual increase	2.1% per year	53% of subregions with annual increase 1.0–4.9%	49% of districts with annual increase 1.0–.4.9%
	Penta1 expected 3% annual increase	0.5% per year	40% of sub-regions with annual increase 1.0–4.9%	28% of districts with annual increase 1.0–4.9%
Consistency between interventions	Penta1 to penta3 ratio: +/− 15% from expected ratio of 1.20	Range from 1.07–1.09 during 2015–2019	80% within plausible range in 2017–2019	75–79% within plausible range in 2017–19
	ANC1 to penta1: +/− 15% from expected ratio of 1.10	Range from 1.00–1.07 during 2015–2019	67–80% within plausible range in 2017–2019	63–71% in plausible range in 2017–19
Denominators	Population projection method (UN): coverage 2017–19 within +/− 10% difference with survey results	ANC1 4% higher; penta1 same as survey; BCG 3% lower than survey	subregions within range for ANC1–43%; penta1–73%; BCG – 67%	districts within range for ANC1–52%, penta1–55%, BCG – 42%

### Completeness and outliers of the reported health facility data

Reporting completeness for RMNCH has been high during 2015–2019, hovering around 98% nationally. The completeness of facility reporting was also above 95% for all years in all subregions, except North Central (94% in 2016) and Kampala (90, 94 and 85% in 2017, 2018 and 2019 respectively). Among the 135 districts, eight districts had reporting rates below 90% in 2018 and in 2019 (Supplementary materials Figure S1). Since reporting rates were high and consistent over time, no adjustments were made for possible service provision by non-reporting facilities which could affect levels and trends. Extreme outliers in the annual data by subregion and district were few. Two extreme outliers for 2015 were corrected, using the expected value rather than the reported value which was likely a data entry error.

### Consistency over time

The national rate of average annual increase for ANC1 numbers was 2.1% during 2015–2019, lower than the expected 3%. Variation over time during 2015–2019 was greater for districts compared to subregions or national level. For ANC1, the annual increase was within the range 1.0–4.9% for eight subregions (53%) and 66 (49%) districts. The average standard error of the regression line, indicative of year-to-year fluctuations, was much greater for districts compared to subregions (0.061 and 0.035, respectively) (Supplementary materials Table S1).

For penta1 reported numbers, there was little increase at the national level (slope of 0.5% per year), mainly due to the higher numbers reported in 2015 and 2016. In six subregions (40%) and 38 districts (28%) the annual increase was within the range of 1.0–4.9%. The standard errors of the regression line were lower at subregional than at district level (mean 0.040 and 0.073 respectively), indicating that annual coverage estimates will be less stable at the district level than at the subregional level.

### Consistency between interventions

The national ratio penta1 to penta3 was almost constant over time at 1.07–1.09 during 2015–2019 (Supplementary materials Table S2). This was lower than the expected ratio based on the UDHS 2016 (1.20), possibly due to overreporting of penta3 overreporting. By subregion, the survey-based penta1/penta3 ratio ranged from 1.07 to 1.34. In 2019, the observed ratio was within a plausible range of the expected ratio in 80% of the 15 subregions, up from 67% in 2015. For the 135 districts, 75% had plausible values in 2019.

The consistency between ANC1 and penta1 reported numbers improved over time (Table S2). The greatest inconsistencies were observed in subregions of Kampala and South Central (all years) and North Central (2015–2018). Kampala had a much higher number of ANC1 visits than penta1 visits, presumably due to women coming to the capital city for services (e.g. for 2019 the ratio was 1.52). South Central region had a major deficit in ANC1 visits compared to penta1 only in one of the 13 districts: Wakiso which accounts for more than half of the population in the South-Central subregion and borders Kampala. This suggests that pregnant women are using ANC services in Kampala (e.g. the ANC1/penta1 ratio in Wakiso in 2019 was 0.80).

### Projected target populations

**Fig. 1:**
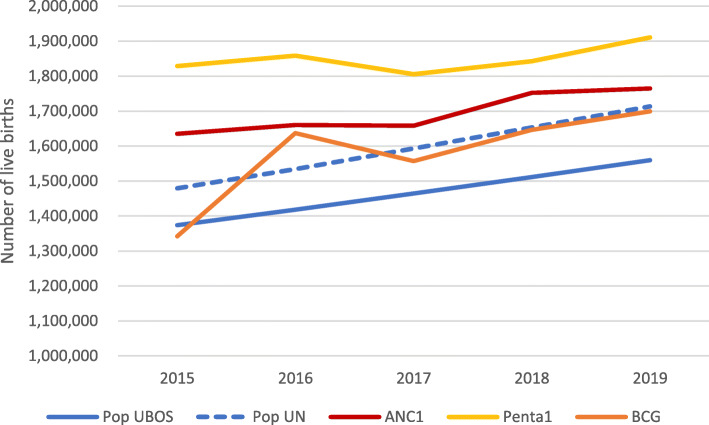
Number of live births, estimated from population projections and from health facility data, 2015–2019

Figure 1 compares the national number of live births for 2015–19, based on five estimation methods. The UBOS population projection with the crude birth rate obtained from the UDHS 2016 results in 1.56 million live births in 2019. Compared to the UN population estimates, there were 1.71 million live births in 2019. The reported numbers of ANC1 (1.76 million) and penta1 (1.91 million in DHIS2) were higher. It is likely that penta1 vaccinations were overreported, as shown above. The estimate of live births based on BCG vaccinations was close to the UN population-based estimate for 2017–2018 but reported numbers in 2015–16 were problematic.

**Fig. 2:**
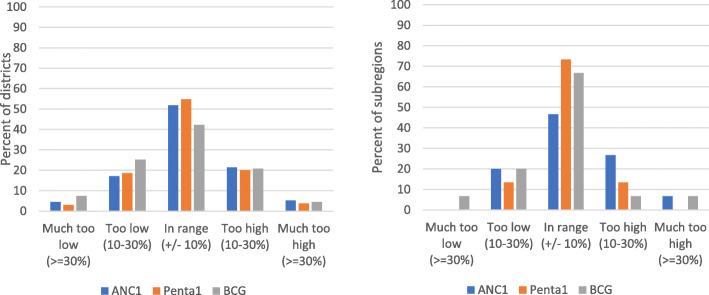
Percent distribution of absolute difference between estimated and expected coverage for ANC1, penta1 and BCG for 2017–2019 (mean), 135 districts (left panel) and 15 subregions (right panel), Uganda

To obtain coverage estimates using population projections for the denominator we used the UN total population estimates, while maintaining the crude birth rate at 3.87 per 1000 population. Figure 2 presents the average difference of the estimated (based on the projected live births) and expected (survey) coverage for ANC1, penta1 and BCG (which should all be above 90%) for the period 2017–2019 for districts (left) and for subregions (right panel). The distribution of the 135 districts shows that just over 50% of districts fall in a +/− 10 percentage points range for penta1 and ANC1 and 42% of districts for BCG. Just under one-tenth of districts had deviations exceeding 30%, and the remaining 40% were in between. Similar proportions were too high (often well over 100%) and too low. The deviations from the expected near universal coverage were greater for districts than for the subregions.

Kampala was an outlier with coverage rates over 150% in all 5 years for ANC1 and also had unlikely high coverage estimates for penta1 and BCG. This implies either severe underestimation of the target population, or issues with the reported number which may be true (service users from other regions/districts) or false (overreporting, but less likely given the consistency over the years).

### Coverage indicators

**Fig. 3:**
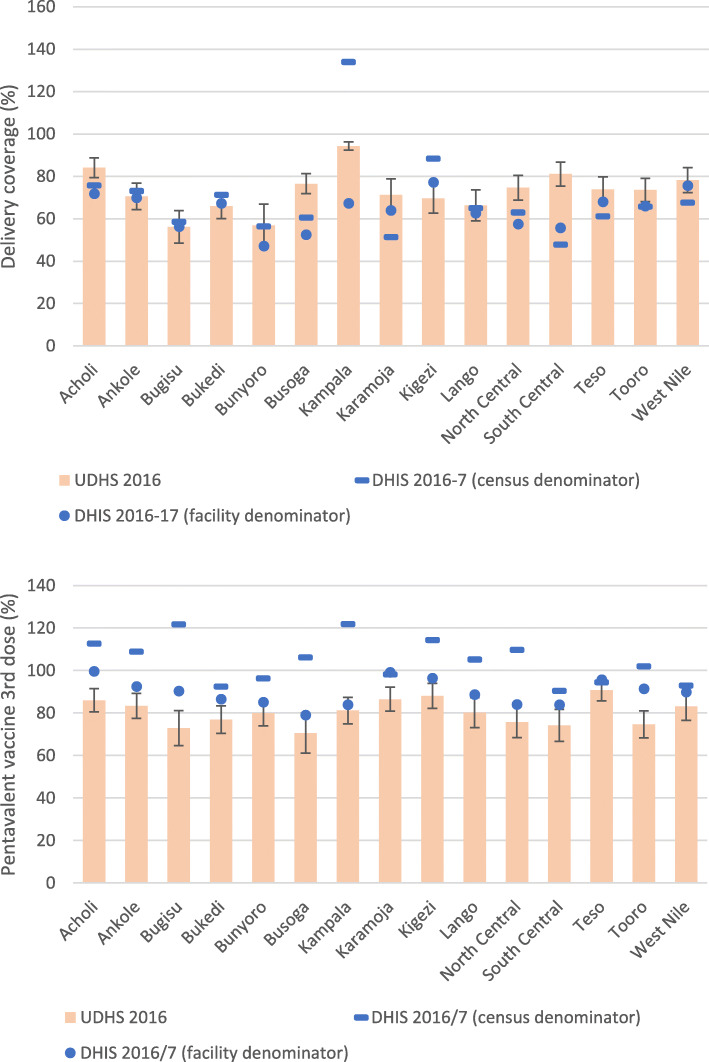
Population coverage of deliveries in health facilities and pentavalent vaccination (third dose) by subregion in the Uganda DHS 2016 (bar with 95% confidence intervals), and coverage derived from health facility reported data according to denominator method (population projection – dash; health facility data derived - dots)

We examined coverage estimates for five indicators, using either population projections or facility data-derived denominators. Figure 3 compares the relative difference between coverage estimates for institutional deliveries and penta3 vaccination by subregion for 2016/17 according to the UDHS 2016 and according to the reported health facility data. For these two indicators the facility-based denominator method performed considerably better than the population projections method. For deliveries, nine subregional estimates fell within the 95% confidence interval of the survey statistic with the facility data method, compared to five subregions with the population projection method. The mean gaps between the UDHS delivery coverage and the facility-based and population projection-based estimates were 16 and 13%, respectively. For penta3 vaccination, the 95% confidence intervals of the survey coverage included three subregions with the facility-based estimate and none with the population projection-based estimate. The mean gaps with the survey coverage were 12 and 31% for the facility and population projection-based methods, respectively. These patterns were also observed for measles coverage, but less so for IPT2 coverage among pregnant women and ANC4 where both denominators performed equally poor compared to the UDHS or UMIS results (Supplementary Figure S2).

The same comparison between the survey statistics and the estimates based on the health facility data using the two denominators was done for districts and showed similar results (Supplementary Figure S3). For many districts, the computed coverage for 2016–17 differed considerably (more than 20%) from the UDHS 2016 value (or UMIS 2018/19 value in case of IPT2). For four of the five indicators, the health facility-based denominator method gave more consistent results than the population projections, especially for the immunization indicators. We also compared year-to-year fluctuations in coverage based on both denominator methods for districts. For most years, the percent of districts with less plausible values is higher for the coverage rates derived from the population projection method compared to the health facility-based denominators.

## Discussion

Routine facility data for monitoring of subnational coverage of MNCH interventions are critical for local and national resource allocation and targeting of interventions. Monitoring of coverage requires good quality reported data on specific interventions as well as accurate target population sizes. Our systematic assessment of the quality of routine reports of key events for RMNCH shows that data quality is a major challenge for the computation of subnational health statistics. On the positive side, Uganda’s completeness of reporting by facilities was near 100% and extreme outliers were rare. On the other hand, major annual fluctuations in reported events and inconsistencies between numbers of reported events were common at district, subregional and national level. Less plausible and inconsistent reported numbers were more common for the 135 districts than for the 15 subregions.

The numbers of reported penta1 vaccinations appeared improbably high – higher than projected population of births or first antenatal visits –, especially in 2015–2016, and even more so for penta3 than for penta1. This may be due to overreporting which has been an issue in other contexts as well, sometimes associated with incentives for vaccinating infants [7, 15, 26].

The estimation of population denominators for coverage estimates is challenging. At the national level, a plausible target population estimates could be made after upward adjustment of the census projections, using the projection by the United Nations Population Division, although several inconsistencies remained. Subnational estimates were more problematic and more so at the district level than the subregional level. Even though working with fixed annual denominators is preferable, also for target setting in districts, the fluctuations in the numbers of reported events rendered large proportions of districts with improbable coverage values within the five-year period. Our analysis showed that an alternative approach based on denominators derived from facility data gives better results but was still unsatisfactory for a substantive number of subregions and districts. This was mainly due to the inconsistencies between the numbers of first antenatal visits and first vaccination, most likely a data quality issue.

The Uganda data illustrate the additional challenges for estimating coverage with health facility data for large urban districts. Kampala and the adjacent district of Wakiso had the greatest inconsistencies. Even though the government health facility reporting system is supposed to cover all types of facilities, there may be challenges with the privately owned facilities. A larger private sector with poorer reporting is often a major factor contributing to such inconsistencies, but also user preferences for the use of, for example, antenatal services in the city of Kampala may play a role. In addition, the quality of reporting of multi-visit indicators, such as ANC and multi-dose vaccinations, may further affect data quality especially in settings where users have multiple choices.

The findings have important implications for efforts to track coverage in individual districts using health facility data. Given the considerable noise in reported events and uncertainty of the target populations, district coverage estimates for MNCH and other health indicators must be interpreted cautiously. More accurate reporting of numerators is critical as it will also help the estimation of target populations, using the near-universal coverage intervention-based methods. This requires greater investments in training and supervision of health workers and data quality control [27–29].

These results also affect the Uganda district league tables where incorrect population denominators lead to systematically over- or under-rating of a district’s performance and where errors in the numerators result in major shifts in district rankings from year to year. The league tables system deals with coverage estimates over 100% by truncating at 100%, but the problem of underestimation of coverage is much harder to detect and correct. In this situation, it may be best to focus on “within reported data” coverage and quality indicators, such as proportion of women who receive ANC4 visits among those who made the first visit or proportion of children who receive measles vaccination among those who received the first pentavalent or BCG vaccination, with or without a correction for non-users of the specific health services. This approach is still highly dependent on the accuracy of reporting by health facilities.

### Limitations

Our approach has several limitations. The external comparison leans heavily on survey-based coverage estimates. The retrospective nature of survey-based estimation, the differences in year of observation with the facility data and sampling errors are all drawbacks on the accuracy of the assumed “gold standard”. On the other hand, our analyses rely heavily on first antenatal visit and first immunizations for which there is little evidence that shows that the coverage rates are not well over 90% almost everywhere in Uganda. We did not have survey-based estimates for districts and used subregional values. Within subregions there may be considerable variation between districts. Furthermore, we did not consider possible declines in fertility, which would imply that the excess reported numbers of near-universal coverage events and expected target populations would even be larger. Population census and surveys however have shown that the fertility decline prior to 2016 was slow.

## Conclusion

Regular reliable estimates of coverage for key MNCH indicators in subnational units including districts are urgently needed for local planning, district monitoring. The quality of routine health facility data however still needs considerable improvement before it can become a reliable instrument for planning, monitoring, and targeting at the district level. In addition, multiple ways of assessing target population sizes for coverage estimates need to be explored, including the use of interventions with potentially near-universal coverage such as first antenatal visit and child immunizations such as first dose of pentavalent vaccination and BCG. Systematic assessment of data quality and transparent adjustments are critical steps towards improving the quality of national and subnational coverage statistics derived from health facility data.

## Supplementary Information

**Additional file 1: Table S1.** Summary of results of regression of reported annual numbers of ANC1 and penta1 by national, subregional and district levels. **Table S2.** Absolute difference between two health facility data coverage estimates with either population projection denominator or health facility data derived denominator and the UDHS 2016 results (or UMIS 2018 in case of IPT2). **Figure S1.** Completeness of reporting for MCH by district, 2015–2019, DHIS2, Uganda. **Figure S2.** Population coverage of measles vaccination among infants, intermittent preventive therapy second dose (IPT 2) among pregnant women and deliveries in health facilities and antenatal care 4th visit by subregion according to Uganda DHS 2016 (bar), and derived from health facility reported data according to denominator method (population projection – dash and health facility data derived - dots). **Figure S3.** Percent of districts with less plausible coverage estimates according to denominator method (population projection and health facility data derived).

## Data Availability

The data used in this study are either publicly available or can be requested directly from the Ministry of Health.

## Abbreviations

ANC1/4: First or fourth antenatal care visit
DHIS2: District Health Information System 2
IPT2: Intermittent preventive therapy second dose
MNCH: Maternal, newborn and child health
Penta1: Pentavalent vaccine (Diphteria, Pertusssis, Tetanus, type b haemophilus influenza, hepatitis B), first dose
UBOS: Uganda Bureau of Statistics
UDHS: Uganda Demographic and Health Survey
UMIS: Uganda Malaria Indicator Survey
