# Supplementary material for: Can routine health facility data be used to monitor subnational coverage of maternal, newborn and child health services in Uganda?
Source: BMC Health Serv Res. 2021 Sep 13;21(Suppl 1):512. doi: 10.1186/s12913-021-06554-6 (PMC8436491; doi:10.1186/s12913-021-06554-6)
Supplement: Supplementary file 1 — Additional file 1: Table S1. Summary of results of regression of reported annual numbers of ANC1 and penta1 by national, subregional and district levels. Table S2. Absolute difference between two health facility data coverage estimates with either population projection denominator or health facility data derived denominator and the UDHS 2016 results (or UMIS 2018 in case of IPT2). Figure S1. Completeness of reporting for MCH by district, 2015–2019, DHIS2, Uganda. Figure S2. Population coverage of measles vaccination among infants, intermittent preventive therapy second dose (IPT 2) among pregnant women and deliveries in health facilities and antenatal care 4th visit by subregion according to Uganda DHS 2016 (bar), and derived from health facility reported data according to denominator method (population projection – dash and health facility data derived - dots). Figure S3. Percent of districts with less plausible coverage estimates according to denominator method (population projection and health facility data derived). [file 12913_2021_6554_MOESM1_ESM.docx]

Supplementary materials

**Table S1: Summary of results of regression of reported annual numbers of ANC1 and penta1 by national, subregional and district levels**

| Indicator |  | National | Subregion | District |
| --- | --- | --- | --- | --- |
| Number |  | 1 | 15 | 135.0 |
| ANC1 | Annual increase 1-4% (N units) | 1 | 8 (53%) | 66 (49%) |
|  | Standard error (average) | 0.015 | 0.035 | 0.061 |
|  | R-squared | 0.87 | 0.47 | 0.61 |
|  |  |  |  |  |
| Penta1 | Annual increase 1-4% | 0 | 6 (40%) | 38 (28%) |
|  | Standard error (average) | 0.022 | 0.04 | 0.1 |
|  | R-squared | 0.121 | 0.504 | 0.4 |

**Table S2**

**Absolute difference between two health facility data coverage estimates with either population projection denominator or health facility data derived denominator and the UDHS 2016 results (or UMIS 2018 in case of IPT2).**

|  | Population projection denominator | | | Population projection denominator | | |
| --- | --- | --- | --- | --- | --- | --- |
|  | Mean difference (%) | Median difference (%) | % of Regions with > 20% difference | Mean difference (%) | Median difference (%) | % of Regions with > 20% difference |
| Deliveries | 16 | 14 | 33 | 13 | 10 | 27 |
| ANC4 | 29 | 27 | 80 | 33 | 31 | 87 |
| Penta3 | 31 | 30 | 80 | 12 | 11 | 13 |
| Measles | 13 | 7 | 27 | 11 | 11 | 7 |
| IPT2 | 14 | 14 | 20 | 17 | 18 | 47 |

**Figure S1: Completeness of reporting for MCH by district, 2015-2019, DHIS2, Uganda.**

**Figure S2**

**Population coverage of measles vaccination among infants, intermittent preventive therapy second dose (IPT 2) among pregnant women and deliveries in health facilities and antenatal care 4^th^ visit by subregion according to Uganda DHS 2016 (bar), and derived from health facility reported data according to denominator method (population projection – dash and health facility data derived - dots).**

**Figure S3**

**Percent of districts with less plausible coverage estimates according to denominator method (population projection and health facility data derived).**
